# Mode of Delivery in the Setting of Repeated Vitreous Hemorrhages in Proliferative Diabetic Retinopathy: A Case Report and Review of the Literature

**DOI:** 10.7759/cureus.11239

**Published:** 2020-10-29

**Authors:** Ahmed M Abdelaal, Abdullah S Alqahtani

**Affiliations:** 1 Ophthalmology, King Abdulaziz Medical City, Ministry of National Guard Health Affairs, Jeddah, SAU; 2 Ophthalmology, King Saud bin Abdulaziz University for Health Sciences, Jeddah, SAU

**Keywords:** proliferative diabetic retinopathy, vitreous hemorrhage, valsalva retinopathy, spontaneous vaginal delivery, pregnancy, cesarean section

## Abstract

The state of pregnancy affects all organ systems including the eyes. Progression of diabetic retinopathy (DR) is a known association. In proliferative DR, there is an increased risk of vitreous hemorrhage (VH) during spontaneous vaginal delivery (SVD) due to the Valsalva maneuver.

A 30-year-old female with poorly controlled type I diabetes and hypothyroidism on treatment was following up with the antenatal services at our hospital. This was her second pregnancy having had a previous miscarriage. Three months into her pregnancy, our Ophthalmology service was consulted to assess her and give our advice regarding the safest mode of delivery for her.

Questioning revealed that she was following regularly elsewhere for proliferate DR with previous interventions and history of multiple and repeated VHs. When she was seen in our Ophthalmology clinic, she was anxious about the mode of delivery that was best suited for her with regard to her ocular condition. On examination, her visual acuity (VA) without correction was 20/40 in both eyes, improving to 20/20 in the right eye and 20/30 in the left eye after refraction. Her intra-ocular pressure was normal. A dilated fundus examination (DFE) showed changes of high-risk proliferative DR in both eyes and a VH in the right eye.

Subsequent follow-up did not reveal any new complaints or concerns. She required one session of pan-retinal photocoagulation (PRP) in her first-trimester visit. DFE showed improvement in VH when compared to her initial examination.

After discussing her condition with her obstetrician, it was decided to offer the patient a cesarean section (C/S) delivery, as her risk of developing VH during SVD was greater than normal. At 38 weeks of gestation, she delivered a healthy boy following an uneventful elective C/S. There were no visual complaints throughout her admission for the procedure or thereafter.

During the reproductive age, DR is a leading cause of decreased vision. Pregnancy is an independent risk factor for progression of DR, with the stage of DR prior to conception being another. If not managed well, proliferative DR can result in VH, with the risk also existing in relation to SVD due to recurrent Valsalva maneuvers during labor. Our patient who initially presented with proliferative DR in both eyes and a VH in the right eye received one session of PRP to both eyes in the first trimester and was closely followed up throughout her pregnancy thereafter. When her due date neared, it was decided that the safest and most suitable mode of delivery was an elective C/S due to her increased risk of VH related to Valsalva maneuvers during SVD, especially since this was to be her first delivery.

## Introduction

Changes that occur during pregnancy affect all organs including the eyes. Risk for progression of diabetic retinopathy (DR) is an example [1]. In the proliferative phase, DR is associated with an increased risk of vitreous hemorrhage (VH) including during spontaneous vaginal delivery (SVD) due to the Valsalva maneuver [1].

The Valsalva maneuver involves an expiratory effort against a closed glottis that increases pressure in the thoracic and abdominal compartments. Valsalva retinopathy (VR) occurs when these increased pressures result in escalations in intraocular venous pressure and rupture of superficial retinal capillaries. Limited studies contribute VR to the efforts of SVD [2,3].

Scarcity of literature and an absence of guidelines hinder standardization of the mode of delivery for women with a history of VH. We report our management of a patient with proliferative DR and repeated VH who delivered by an elective cesarean section (C/S) following consensus between her obstetrician and ophthalmologist. We also review the related literature.

## Case presentation

A 30-year-old female with poorly controlled type I diabetes and hypothyroidism on treatment was following with the antenatal services at our hospital. This was her second pregnancy having had a previous miscarriage. Three months into her pregnancy, our Ophthalmology service was consulted to assess her and give our advice regarding the safest mode of delivery for her.

Ophthalmic history revealed that she underwent phacoemulsification with intra-ocular lens implantation in both eyes and was following up regularly elsewhere for proliferative DR. Her condition required interventions in the form of anti-VEGF (vascular endothelial growth factor) injections once in both eyes, two years before becoming pregnant. She also received three sessions of pan-retinal photocoagulation (PRP) to both eyes, the last being seven months prior to conception. There was a history of multiple and repeated VHs.

**Figure 1:**
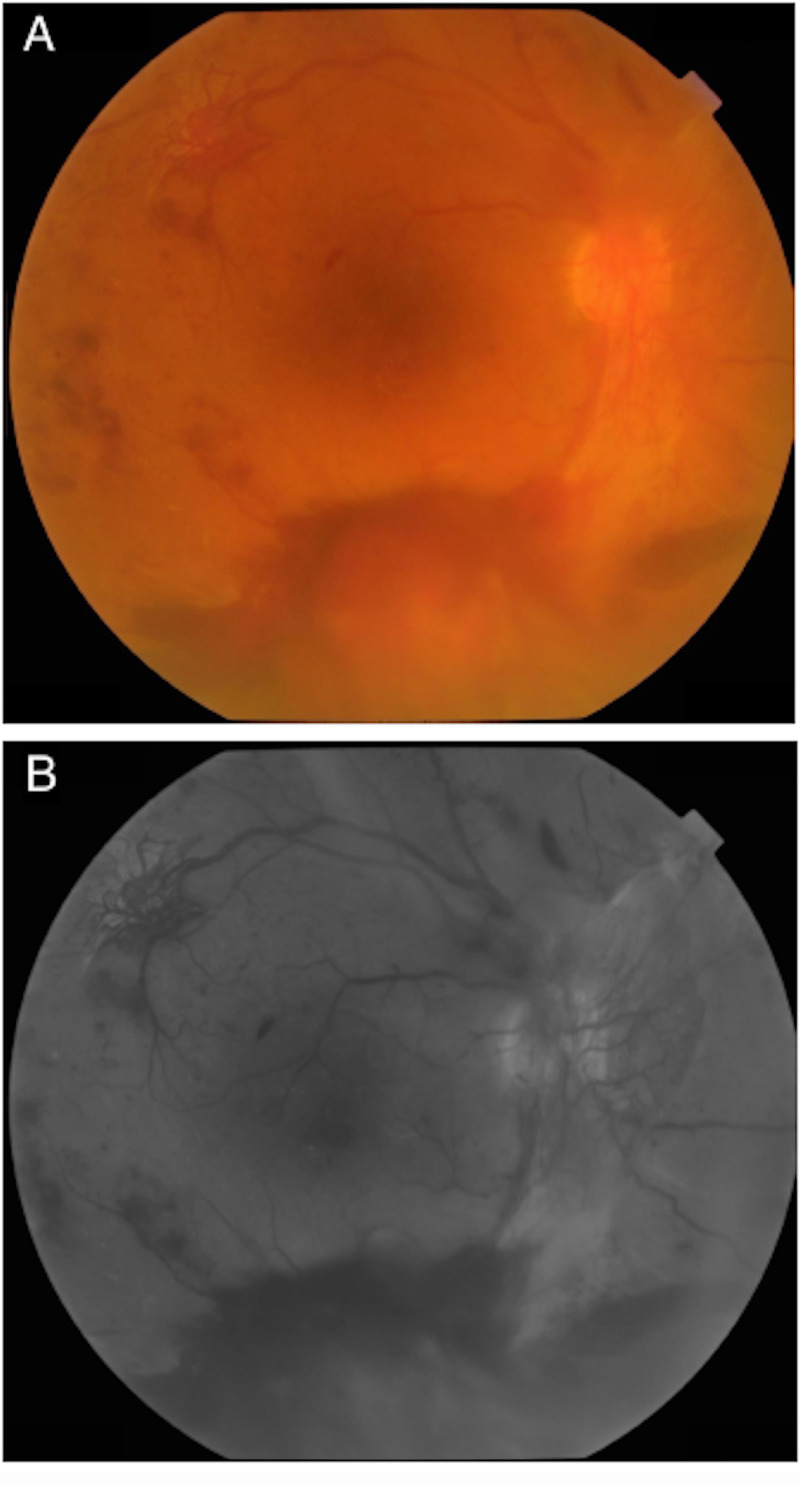
Right eye fundus photos: (A) colored and (B) red free Fundus photos of the right eye showing high-risk proliferative diabetic retinopathy with inferior vitreous hemorrhage, neovascularization of the optic disc, and neovascularization elsewhere involving the superior temporal arcade.

**Figure 2:**
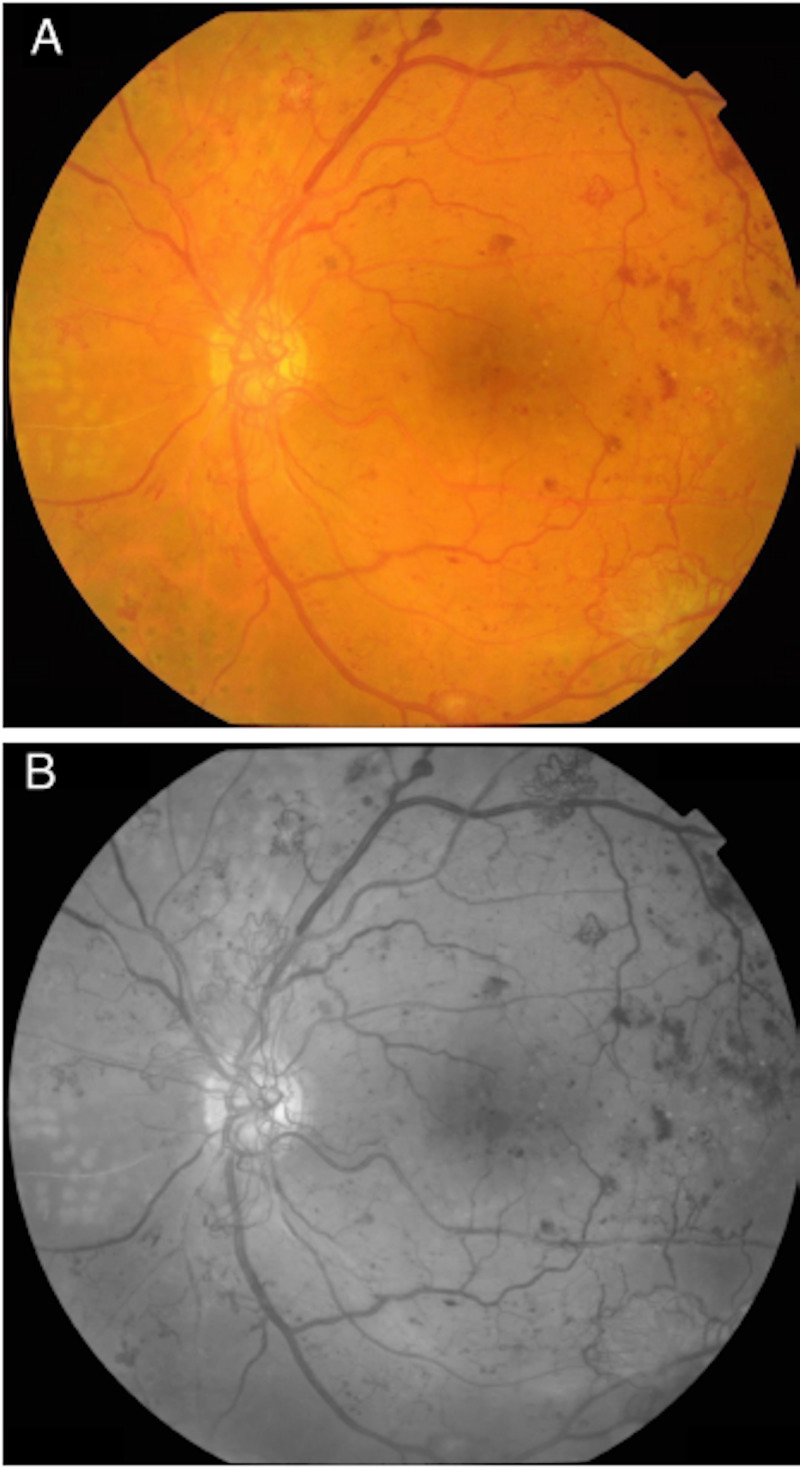
Left eye fundus photos: (A) colored and (B) red free Fundus photos of the left eye showing high-risk proliferative diabetic retinopathy with neovascularization of the optic disc and neovascularization of the superior and inferior temporal arcades with scattered exudates and blot hemorrhages temporally.

When she presented to our Ophthalmology clinic, she was anxious about her condition in relation to the mode of delivery she should undergo. On examination, her visual acuity (VA) without correction was 20/40 in both eyes, improving to 20/20 in the right eye and 20/30 in the left eye with refraction. Her intra-ocular pressure (IOP) was normal in both eyes. Examination of her anterior segments showed bilateral pseudophakia and the absence of iris neovascularization in either eye. A dilated fundus examination (DFE) showed changes of high-risk proliferative DR. Colored fundus photos (Figures 1A, 2A) and red-free fundus photos (Figures 1B, 2B) were obtained for both eyes. There was an inferior VH in the right eye, neovascularization of the disc in both eyes, neovascularization involving the posterior pole of both eyes, and scattered PRP marks peripherally in both eyes.

In the subsequent three follow-up visits during the last trimester, she had no complaints or active issues. Her VA ranged between 20/40 to 20/60 in the right eye and 20/30 to 20/40 in the left eye without correction, improving after refraction to a maximum of 20/20 and 20/25 in the right and left eyes, respectively. Her IOP remained normal. DFE showed improvement in VH when compared to her earlier examination and the presence of regressing high-risk proliferative DR and PRP marks in both eyes.

Following discussion with her Obstetrician and due to her history of repeated VHs and examination findings, it was decided to offer the patient a C/S delivery, as her risk of developing VH during SVD was greater than normal, especially as this would be her first delivery with expectations of prolonged labor and extended periods of raised intra-thoracic and intra-abdominal pressure and consequent retinal capillary pressures. At 38 weeks of gestation, she delivered a healthy boy following an uneventful elective C/S. There were no visual complaints throughout her admission for the procedure or thereafter.

She was seen four months postpartum, and at that time she denied any visual complaints since the time of delivery. Her VA was stable at 20/40 in the right eye and 20/60 in the left eye without correction and improved with refraction to 20/20 and 20/25 in the right and left eyes, respectively. Her IOP remained normal, and her DFE showed a flat fibrotic retina corresponding to moderate proliferative DR. The plan was to closely follow her up without surgical intervention unless VA deteriorates.

## Discussion

DR is a leading cause of decreased vision in patients between 20 and 64 years of age, the range which encompasses the reproductive years women are most likely to become pregnant [4]. Pregnancy is an independent risk factor for hastening progression of DR [5]. The stage of DR prior to conception is another risk factor [1,5]. Proliferative DR if not adequately managed can result in VH, and the risk also exists during SVD due to the series of Valsalva maneuvers during labor [1]. Our patient required multiple ocular interventions prior to her presentation to our antenatal services due to her high-risk proliferative DR in both eyes and multiple VHs in the right eye.

VR occurs when the increased pressures during Valsalva maneuvers lead to sudden escalations in intraocular venous pressure and consequent rupture of superficial retinal capillaries, especially in long-standing DR where the retinal vessels are more fragile. Patients who experience VR usually give a history of sudden loss of vision following a bout of heavy lifting, straining, coughing, or vomiting [3]. It may even occur during sleep where the cerebral and retinal vessels are under higher pressures than when in the standing position. Limited studies also attribute VR to SVDs [2,3]. Based on the pathophysiology behind VR and reported cases, one may consider elective C/Ss to be safer than SVDs in women with a history of retinal or VH or those with higher risks than usual.

In those with proliferative DR or severe non-proliferative DR, vigorous aerobic or resistance exercise may be contraindicated during pregnancy according to the American Diabetes Association due to the risk of precipitating VH or retinal detachment [6]. The concern is that during these exercises, which involve repeated Valsalva maneuvers, hemorrhagic thresholds that precipitate intra-retinal hemorrhage, pre-retinal hemorrhage, and/or VH may be exceeded [7]. Since SVDs include powerful, repetitive Valsalva maneuvers, it is assumed to be risky in women with severe non-proliferative or proliferative DR, leading to suggestions that C/Ss can minimize the risk of VH associated with SVDs [8].

However, C/Ss require epidural anesthesia, which has been reported to precipitate retinal or VH in rare cases [9,10]. One of the hypotheses was the Trendelenburg position could increase intraocular venous pressure during labor and the likely occurrence of a Valsalva maneuver [9]. The other proposition was that the use of epidural anesthesia resulted in increased cerebrospinal fluid pressure and consequently increased retinal venous pressure, leading to retinal hemorrhages in their cases [10].

Without standards for obstetricians to manage patients with even the most dreaded and sight-threatening ophthalmic conditions, patients will continue to be managed based on personal opinions and experience rather than concrete evidence. A study was conducted surveying an international audience of obstetricians regarding their opinions on the mode of delivery they would implement for women with previous retinal detachment surgery to prevent re-detachment from occurring. The majority (76%) chose assisted delivery (either C/S or instrumental delivery), with 58% basing their decision on personal opinion and not on existing guidelines or literature. Intriguingly, only 13% would have sought opinion of an ophthalmologist [11].

For cases similar to ours, we recommend PRP during pregnancy when required and eventual C/S delivery for primigravidas. If macular edema is present, steroid injections may be used, but anti-VEGF agents should be avoided due to the risk of teratogenicity.

## Conclusions

The absence of guidelines for obstetricians in choosing the best mode of delivery in the setting of ophthalmic disease necessitates close cooperation between obstetricians and ophthalmologists for deciding the best modality of delivery for patients individually when complications are present.
